# Biocompatibility Evaluation of Carbon Nanohorns in Bone Tissues

**DOI:** 10.3390/nano13020244

**Published:** 2023-01-05

**Authors:** Katsuya Ueda, Chuang Ma, Makoto Izumiya, Chika Kuroda, Haruka Ishida, Takeshi Uemura, Naoto Saito, Kaoru Aoki, Hisao Haniu

**Affiliations:** 1 Biomedical Engineering Division, Graduate School of Medicine, Science and Technology, Shinshu University, 3-1-1 Asahi, Matsumoto 390-8621, Nagano, Japan; 2 Department of Organ Anatomy and Nanomedicine, Graduate School of Medicine, Yamaguchi University 1-1-1 Minami-Kogushi Ube, Yamaguchi 755-8505, Japan; 3 Institute for Biomedical Sciences, Interdisciplinary Cluster for Cutting Edge Research, Shinshu University, 3-1-1 Asahi, Matsumoto 390-8621, Nagano, Japan; 4 Division of Gene Research, Research Center for Advanced Science, Shinshu University, 3-1-1 Asahi, Matsumoto 390-8621, Nagano, Japan; 5 Physical Therapy Division, School of Health Sciences, Shinshu University, 3-1-1 Asahi, Matsumoto 390-8621, Nagano, Japan; 6 Department of Orthopedic Surgery, School of Medicine, Shinshu University, 3-1-1 Asahi, Matsumoto 390-8621, Nagano, Japan

**Keywords:** nanoparticles, carbon nanohorns, carbon blacks, osteoclast-like cells, osteoblast-like cells, biocompatibility, bone regeneration, drug delivery system

## Abstract

With the advent of nanotechnology, the use of nanoparticles as drug delivery system (DDS) has attracted great interest. We aimed to apply carbon nanohorns (CNHs) as DDS in the development of new treatments for bone diseases. We evaluated the in vitro and in vivo cellular responses of CNHs in bone-related cells compared with carbon blacks (CBs), which are similar in particle size but differ in surface and structural morphologies. Although in vitro experiments revealed that both CNHs and CBs were incorporated into the lysosomes of RAW264-induced osteoclast-like cells (OCs) and MC3T3-E1 osteoblast-like cells (OBs), no severe cytotoxicity was observed. CNHs reduced the tartrate-resistant acid phosphatase activity and expression of the differentiation marker genes in OCs at noncytotoxic concentrations, whereas the alkaline phosphatase activity and differentiation of OBs increased. Under calcification of OBs, CNHs increased the number of calcified nodules and were intra- and extracellularly incorporated into calcified vesicles to form crystal nuclei. The in vivo experiments showed significant promotion of bone regeneration in the CNH group alone, with localized CNHs being found in the bone matrix and lacunae. The suppression of OCs and promotion of OBs suggested that CNHs may be effective against bone diseases and could be applied as DDS.

## 1. Introduction

With the progress of nanotechnology, new nanomaterials have been developed and applied across a wide range of disciplines, ranging from industrial to medical fields [1,2]. In recent years, nanomedicine has emerged as a promising approach to medicine [3], and nanomaterials have attracted a great deal of interest for their potential use as drug delivery systems (DDSs). A variety of nanoparticles (NPs) have been studied as DDS, including protein constructs, lipid micelles, ribonucleic acid, superparamagnetic iron oxide NPs, and gold nanoclusters [4].

Many studies have been conducted on carbon nanomaterials (CNMs) regarding their application in the living body. Because of the large specific surface area and ease of modification in CNMs such as carbon nanotubes (CNTs) and fullerene, these nanomaterials can potentially carry anticancer drugs and have been used as DDS for tumors [5,6,7]. One of these CNMs, carbon nanohorns (CNHs), are graphene sheet tubes in which one end is closed. The cylindrical structure has a length of 40–50 nm and diameter of 2–5 nm, and thousands of CNH are radially assembled to form a spherical aggregate with a diameter of approximately 100 nm. The aggregate presents a unique mechanism that enables the encapsulation of drugs [8,9,10,11].

Various studies have started to explore the use of CNHs as DDS. One previous study has reported the low toxicity of CNHs in vivo using rabbits [12]. Murakami et al. [13] used dexamethasone on the surface of CNHs and evaluated their drug efficacy in vitro. In addition, Ajima et al. [10,11] reported on the encapsulation mechanism of CNH and found that the local administration of the anticancer drug cisplatin encapsulated inside CNH in mice resulted in its effective application as DDS.

We have previously reported the possibility that CNTs, which have a tubular structure similar to CNHs, may be effective for treating bone diseases [14,15]. We are currently investigating CNHs as DDSs in the field of bone tissue regeneration. We have also reported the possibility of combining an anti-osteoclastic drug with CNHs using calcium phosphate and incorporating the aggregate into lysosomes to effectively suppress RAW264-induced osteoclast-like cells (OCs). This aggregate can be applied as a localized DDS for treating bone metastasis [14,15]. Many studies have evaluated the cellular responses of CNHs to macrophages [16,17], which are the progenitor cells of osteoclasts. We reported that macrophages can recognize the surface morphology of nanomaterials and differ in their various responses, such as cellular uptake [18]. Furthermore, CNHs have been reported to be phagocytosed by macrophages, which produce substances that promote bone regeneration [19,20]. In addition, one study reported the promotion of bone formation by coating the surface of titanium with CNHs [21]. Although numerous studies have described the application of CNHs as DDS in bone tissues, few reports have evaluated their direct effect on bone tissues.

In the present study, we aimed to clarify the biological characteristics of CNHs alone as DDS for new treatment of bone diseases. In addition, we evaluated the bio-response to bone-related cells via in vitro and in vivo experiments. Furthermore, to evaluate the effect of morphological differences, we compared CNHs and carbon blacks (CBs) of similar particle sizes that differed in terms of surface and structural morphologies.

## 2. Materials and Methods

### 2.1. Sterilization of CNMs

CNHs with an aggregate diameter of approximately 100 nm, purity of 95% or more, graphene content of less than 5%, and iron content of 0% were used (CNHox; NEC, Tokyo, Japan). CBs with a diameter of approximately 120 nm were used (Asahi#8, Asahi Carbon Co., Ltd., Niigata, Japan). These CNMs were sterilized in an autoclave at 121 °C for 20 min and dried before use.

### 2.2. Preparation of Highly Dispersed CNMs

Bovine serum albumin (Sigma, St. Louis, MO, USA) was dissolved to a concentration of 10 mg/mL in Hanks’ balanced salt solution (Nacalai Tesque, Kyoto, Japan) with filter sterilization to create a dispersing agent. Sterilized CNMs were subsequently dissolved to a concentration of 10 mg/mL with the dispersant, sonicated for 60 min with a PR-1 sonicator (Thinky, Tokyo, Japan), and used as a base sample for each experiment. The rheological size and zeta potential of the dispersed CNMs were measured using a Zetasizer Nano ZS (Malvern Instruments, Malvern, UK; Table 1). Each dispersant was adjusted to a concentration of 100 µg/mL and measured three times.

### 2.3. Cell Cultures

The murine macrophage-like cell line RAW264 was purchased from Riken BRC (Ibaraki, Japan). RAW264 cells were cultured in a minimum essential medium (MEM; Nacalai Tesque) with 10% fetal bovine serum (FBS; Sigma), nonessential amino acid solution (×100; Nacalai Tesque), and penicillin-streptomycin-amphotericin B suspension (×100; FUJIFILM Wako, Osaka, Japan). Murine calvaria-derived osteoblast-like cell line (OBs) MC3T3-E1 was also purchased from Riken BRC. MC3T3-E1 cells were cultured in a medium containing αMEM (Nacalai Tesque) supplemented with 10% FBS and penicillin-streptomycin-amphotericin B suspension. Both cells were passaged twice a week and maintained at 37 °C and 5% CO_2_.

### 2.4. Cell Viability Evaluation of CNMs in OCs and OBs

OCs were prepared using the following procedure in all experiments: RAW264 cells were seeded first in a 96-well plate at a density of 3.1×10^3^ cells/cm^2^ and cultured in αMEM supplemented with 10% FBS and penicillin-streptomycin-amphotericin B suspension. Twenty-four hours after incubation, a receptor activator of nuclear factor kappa-B ligand (RANKL; Oriental Yeast Co., Ltd., Tokyo, Japan) was added to the cultured medium at a concentration of 200 ng/mL and further incubated for 4 days.

OBs were seeded in a 96-well plate at a density of 3.1 × 10^4^ cells/cm^2^. After 24 h, CNMs were added to the cultures and incubated for 24 or 48 h. The cell viability was analyzed using alamarBlue® reagent (Bio-Rad, Hercules, CA, USA) according to the manufacturer’s instructions. Briefly, the alamarBlue® reagent was diluted in the medium (1:10) and added to the cultures. After 1 h of incubation, fluorescence intensity was measured using a PlateReader AF2200 (Eppendorf, Hamburg, Germany) with the excitation/emission wavelengths set at 535/590 nm. The group to which only the dispersant was added was designated as the control, and cell viability was calculated with the control set as 100%.

### 2.5. Evaluation of CNM Uptake in OCs and OBs

OCs and OBs were seeded and cultured in µ-Slide 8 wells (ibidi GmbH, Lochhamer Schlag, Germany) under the same conditions described in Section 2.4. Twenty-four hours after CNM exposure, the cultures were washed twice with Dulbecco’s phosphate-buffered saline (DPBS; Nacalai Tesque) and subsequently stained with a nuclear staining solution (bisbenzimide H33342 fluorochrome trihydrochloride dimethyl sulfoxide solution; Nacalai Tesque) and lysosome staining solution (CytoPainter lysosomal staining ab138895; Abcam, Cambridge, UK) for 30 min. The cells were then washed twice with DPBS. The images were acquired using a BZ-X710 inverted fluorescent phase-contrast microscope (Keyence, Osaka, Japan).

### 2.6. Subcellular Localization of CNMs in OCs and OBs

A sterilized cover slip was placed in a 48-well plate, and the cells were cultured under the same conditions as in Section 2.4. After culturing, the cells were exposed to 5 µg/mL of CNHs or CBs. After 24 h, the cultures were washed with phosphate buffer (PB) and fixed with 2.5% glutaraldehyde. The fixed cultures were post-fixed with a 1% osmium tetroxide solution, dehydrated, and embedded in epoxy resin. Ultrathin sections were prepared, and electron micrographs were obtained with an electron microscope (TEM; JEM-1400HC, JEOL Ltd., Tokyo, Japan).

### 2.7. Effect of CNMs on TRAP/ALP Activity

OCs were seeded in a 24-well plate at a density of 3.1 × 10^3^ cells/cm^2^ and exposed to 5 µg/mL of CNHs or CBs for 4 days. OBs were similarly seeded in a 24-well plate at a density of 3.1 × 10^3^ cells/cm^2^ and exposed to 5 µg/mL of CNHs or CBs for 5 days.

The tartrate-resistant acid phosphatase (TRAP) activity in the OC lysate and alkaline phosphatase (ALP) activity in OB lysate were analyzed using a TRACP/ALP assay kit (Takara Bio, Shiga, Japan) with a PlateReader AF2200 according to the manufacturer’s protocol. The medium was removed after culturing, and the cells were washed twice with a normal saline solution. The cells were lysed with saline solution containing 1% NP-40 (Nacalai Tesque) and centrifuged at 2000× *g* and 4 °C for 30 min. According to the manufacturer’s protocol, the TRAP bioactivity of OCs and ALP bioactivity of OBs were used to measure absorbance at 405 nm with a PlateReader AF2200 after 60 min and 30 min, respectively. The protein concentrations of both OCs and OBs were measured using a bicinchoninic acid assay kit (Nacalai Tesque). The TRAP activity of OCs and ALP activity of OBs were both expressed as absorbance at 405 nm/protein weight (g).

### 2.8. Effects of CNMs on the Gene Expression of OCs and OBs

OCs and OBs were seeded in a 6-well plate at 3.1 × 10^3^ cells/cm^2^ and exposed with 5 µg/mL CNHs or CBs for 4 and 5 days, respectively. The total RNAs were extracted using an RNA extraction kit (Nippon Genetics, Tokyo, Japan) according to the manufacturer’s protocol. ReverTra Ace qPCR RT Master Mix with gDNA Remover (Toyobo, Osaka, Japan) was used for cDNA synthesis. Real-time PCR was performed using THUNDERBIRD Next SYBR^®^ qPCR Mix (Toyobo) with Step One Plus Real-Time PCR System (Thermo Fisher Scientific, Waltham, MA, USA). The target genes and primers (Takara Bio) are shown in Table 2.

### 2.9. Assessment of the Effects of Calcification on OBs on CNMs

OBs were seeded in a 48-well plate at a density of 3.1 × 10^3^ cells/cm^2^ and incubated for 24 h. Then, 100 µg/mL of vitamin C (Nacalai Tesque) and 5 mM of β-glycerophosphate (Calbiochem, LaJolla, CA, USA) were added to the culture medium. CNMs were diluted to a concentration of 5 µg/mL in a calcified medium supplemented with CNMs and cultured for 28 days. Half of the medium was replaced with fresh calcified medium every 3 days. The cells were centrifuged at 12,000× *g* for 10 min at 4 °C. The supernatant was aspirated without removing CNMs, suspended in fresh calcification medium, and returned to the original wells. After culturing for 28 days, the cells were washed and fixed with ice-cold methanol for 20 min, washed again, and stained with 1% alizarin red S (Sigma; pH 6.4) for 5 min to evaluate bone mineralization.

### 2.10. Evaluation of Intracellular Uptake of CNMs in OBs under Calcified Conditions

After culturing for 28 days, the cells were washed with PB and fixed with 2.5% glutaraldehyde. The fixed samples were post-fixed with a 1% osmium tetroxide solution, dehydrated, and embedded in epoxy resin. Ultrathin sections were prepared and observed with a TEM.

### 2.11. Evaluation of Bone Regeneration in Rat Tibia

The animal protocol was approved by the Committee for Animal Experiments of Shinshu University (Approval No. 019072). Male rats (Wistar, 10 weeks old) were anesthetized with a mixed anesthetic (medetomidine, midazolam, and butorphanol), and both tibiae were reamed with an 18G syringe needle to remove the bone. Using a microsyringe, 50 μL of the solution was injected into the defect site at a concentration of 10 mg/mL for each sample. The bone tissue structures were observed immediately postoperatively and 12 weeks postoperatively using µ-CT (Latheta LCT-200; Hitachi, Ltd., Tokyo, Japan). The rats were euthanized using carbon dioxide gas after sufficient bone regeneration had been observed, and the tibiae were then excised. The samples were fixed with 4% paraformaldehyde and decalcified with EDT-X decalcifying solution (Pharma Co., Ltd., Tokyo, Japan). After decalcification, each sample was sliced, and bone tissue specimens were prepared. Tissue slices were stained with hematoxylin eosin (HE) and Masson’s trichrome (MT) to evaluate bone tissue regeneration. In addition, we quantified the amount of newly formed bone using bone histology around the defect, where most bone regeneration was observed, using ImageJ software (Ver. 2.9.0).

### 2.12. Statistical Analysis

Statistical analysis was performed using BellCurve for Excel (Social Survey Research Information Co., Ltd., Tokyo, Japan). Statistical significance was evaluated using the Steel–Dwass test or one-way ANOVA followed by the post hoc Tukey–Kramer or Dunnett test. Data are represented as mean ± SD or boxplots, as indicated in the figure legends. Statistical significance was assumed when *p* < 0.05.

## 3. Results

### 3.1. Effect of CNMs on Cell Viability of OCs and OBs

**Figure 1:**
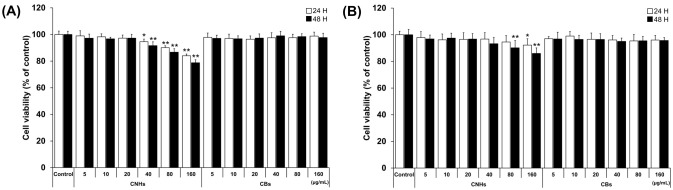
Effect of CNMs on the cell viability of OCs and OBs. (**A**) Cell viability of OCs exposed to CHNs and CBs. Cell viability was analyzed after 24 and 48 h of exposure. n = 5 cultures (**B**) Cell viability of OBs exposed to CHNs and CBs. Cell viability was analyzed after 24 and 48 h of exposure. n = 5 cultures. Control cultures were exposed to dispersants without CNMs. Data are represented as mean ± SD. Statistical significance was evaluated using Dunnett’s multiple comparison test. * *p* < 0.05 and ** *p* < 0.01, compared with the control.

We first evaluated the biocompatibility of CNHs together with CBs using relevant cells of bone tissue to assess whether CNHs could be a DDS for bone tissue. To examine the cytotoxic effect of CNHs, different concentrations of CNHs were applied to the OCs and OBs. The cell viability of OCs was not affected by low concentrations of CNHs (5, 10, and 20 μg/mL). However, at higher concentrations of CNHs (40, 80, and 160 μg/mL), the cell viability was decreased in a concentration-dependent manner (Figure 1A). On the other hand, a slight decrease in cell viability was observed in OB cells when exposed to high concentrations of CHNs (80 and 160 μg/mL) (Figure 1B). In contrast to CNHs, OBs did not affect the cell viability of OCs and OBs. As shown in these results, the reduction in cell viability of OCs and OBs was not cell death, but only a slight inhibition of cell proliferation at high concentrations.

### 3.2. Intracellular Uptake of CNMs in OCs and OBs

**Figure 2:**
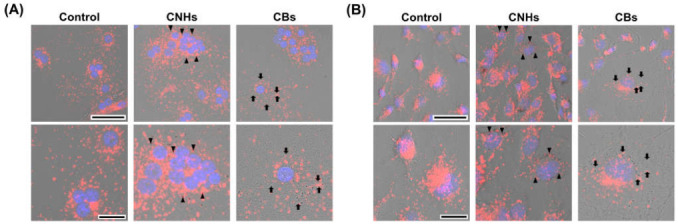
Intracellular uptake of CNMs into OCs and OBs. (**A**) Intracellular uptake of CNHs and CBs in OCs. The cultures were exposed to CNHs and CBs for 24 h. Control cultures were exposed to dispersants without CNMs. (**B**) Intracellular uptake of CNHs and CBs in OBs. The cultures were exposed to CNHs and CBs for 24 h. Control cultures were exposed to dispersants without CNMs. The cells were stained with nuclear staining (blue) and lysosomal staining (red), and phase-contrasted-merged images are shown. High magnification images are shown in the lower panels. Arrows and arrowheads indicate intracellular CNHs and CBs, respectively. Scale bars: 50 μm (**upper panels** in A and B) and 20 µm (**lower panels** in A and B).

**Figure 3:**
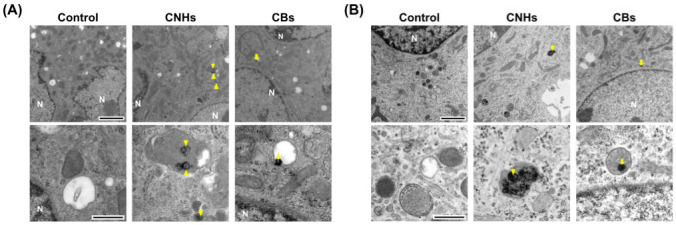
Subcellular localization of CNM uptake. (**A**) Subcellular localization of CNHs and CBs taken up into OCs. (**B**) Subcellular localization of CNHs and CBs taken up into OBs. The cultures were exposed to CNHs and CBs for 24 h. N, nuclei. Arrows and arrowheads indicate intracellular CNHs and CBs, respectively. High magnification images are shown in the lower panels. Scale bars: 2 μm (**upper panels** in A and B) and 500 nm (**lower panels** in A and B).

Since it was important to know whether the DDS carriers were taken up by the target cells, we next observed whether CNHs were taken up by bone-related cells. Fluorescence microscope images of OCs and OBs showed that both CNH and CB signals were well-merged with lysosomal staining signals (Figure 2). Furthermore, the TEM images confirmed that highly dispersed CNHs and CBs were localized within the structure of the lysosomes (Figure 3). These results suggest that CNMs are taken up by these cells and that CNMs are encapsulated in lysosomes. In addition, there was no difference in subcellular localization between CNHs and CBs in either cell, but their number was observed to be higher in CNHs.

### 3.3. Effect of CNHs on TRAP/ALP Activity of OCs and OBs

**Figure 4:**
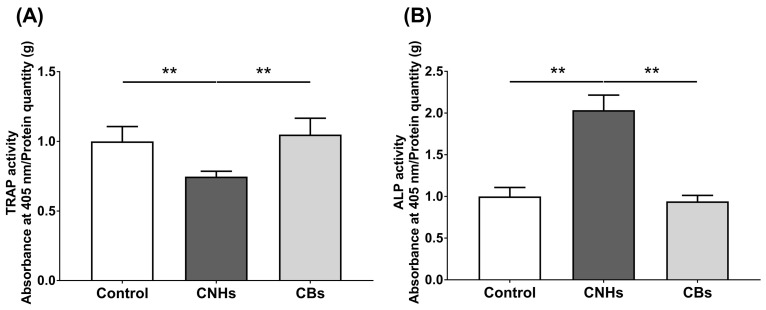
TRAP/ALP activity of CNMs exposed to OCs and OBs. (**A**) TRAP activity of OCs exposed to CNHs and CBs. n = 8 cultures. (**B**) ALP activity of OBs exposed to CNHs and CBs. n = 8 cultures. Data are represented as mean ± SD. Statistical significance was evaluated using the Tukey–Kramer test. ** *p* < 0.01.

To determine how the intracellular uptake of CNHs affects the bioactivity of bone-related cells, we examined the enzymatic activity of OCs and OBs markers by measuring the TRAP/ALP activity. The TRAP activity in lysate prepared from CNH-exposed OCs was significantly decreased by approximately 30% compared with that from the control OCs (Figure 4A). In contrast, ALP activity was significantly increased by approximately 200% in lysate prepared from CNH-exposed OBs (Figure 4B). CBs did not affect the TRAP/ALP activities in these cells. These results suggest that CNHs can exert different effects on the differentiation of OCs and CBs.

### 3.4. Effect of CNHs on Differentiation-Related Genes of OCs and OBs and Cytokine Genes

**Figure 5:**
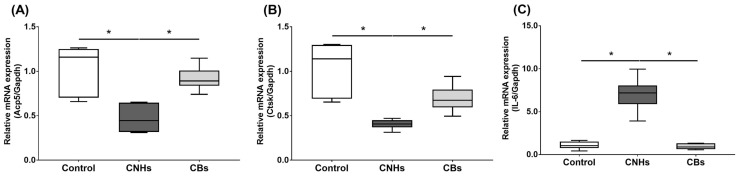
Effects of CNMs on gene expression in OCs. The OCs were exposed to CHNs and CBs. The relative amounts of *Acp5* (**A**), *Ctsk* (**B**), and *IL-6* (**C**). mRNAs were analyzed using quantitative real-time PCR (n = 6 cultures each). The horizontal line in each box indicates the median, the box shows the interquartile range (IQR), and the whiskers are 1.5 × IQR. Statistical significance was evaluated using the Steel–Dwass test. * *p* < 0.05.

**Figure 6:**
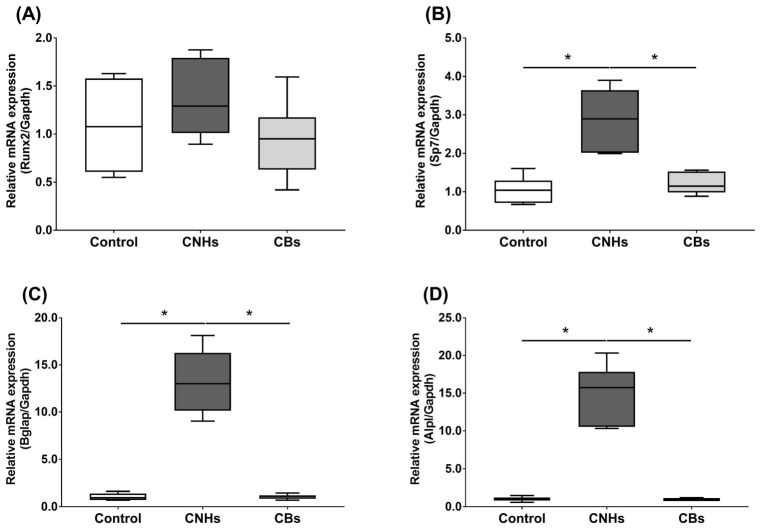
Effects of CNMs on gene expression in OBs. The OBs were exposed to CHNs and CBs. The relative amounts of *Runx2* (**A**), *Sp7* (**B**), *Bglap* (**C**), and *Alpl* (**D**). mRNAs were analyzed by quantitative real-time PCR (n = 6 cultures each). The horizontal line in each box indicates the median, the box shows the interquartile range (IQR), and the whiskers are 1.5 × IQR. Statistical significance was evaluated using the Steel–Dwass test. * *p* < 0.05.

Next, we not only examined the enzymatic activity, but also the effect of CNHs taken up into the cells at the level of gene expression using differentiation markers in OCs and OBs. To examine the effects of CNHs on the expression of differentiation-related genes, we performed real-time quantitative RT-PCR. The OCs exposed to CNHs showed a decrease in the amount of Acp5 and Ctsk mRNA. These osteoclast differentiation-related genes decreased to approximately 40% of those in the control cells (Figure 5A,B). In these cells, proinflammatory cytokine IL-6 mRNA was dramatically increased compared with that in the control cultures (Figure 5C). On the other hand, CBs did not affect the expression of these genes. The exposure to CNH significantly increased the amount of Sp7 mRNA, which is a downstream transcription factor of Runx2 in OBs (Figure 6B). Exposure to CNH slightly but not significantly increased the amount of an early differentiation transcription factor Runx2 mRNA in OBs (Figure 6A). In these cells, Bglap and Alpl mRNA were dramatically increased compared with those in the control cells (Figure 6C,D). CBs did not affect the expression of these genes.

### 3.5. Effect of CNHs on OBs under Calcified Conditions

**Figure 7:**
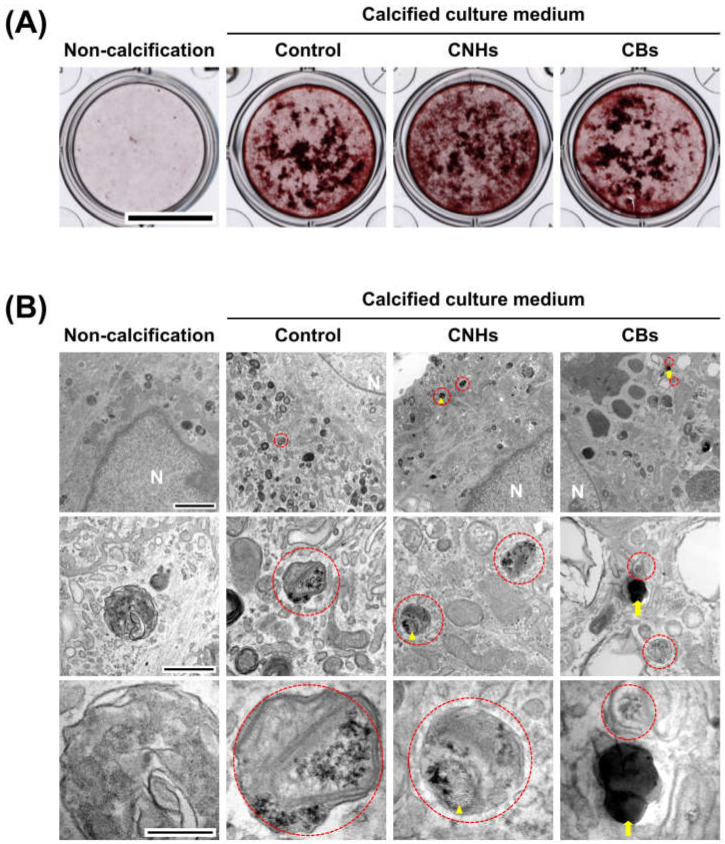
Effect of CNMs on OB calcification. (**A**) Effect of CNHs and CBs on the calcification of OBs. OBs were exposed to CNHs and CBs and calcified by calcified culture medium containing vitamin C and β-glycerophosphate. Scale bars, 10 mm. (**B**) Transmission electron microscope images of OBs. OBs were exposed to CNHs and CBs and calcified under the same conditions in (**A**). Enclosed dashed lines indicate calcified vesicles. Arrows and arrowheads indicate intracellular uptake of CNHs and CBs, respectively. Different magnification images are shown. Scale bars: 2 μm (**upper** panels), 500 nm (**middle** panels), and 200 nm (**lower** panels).

**Figure 8:**
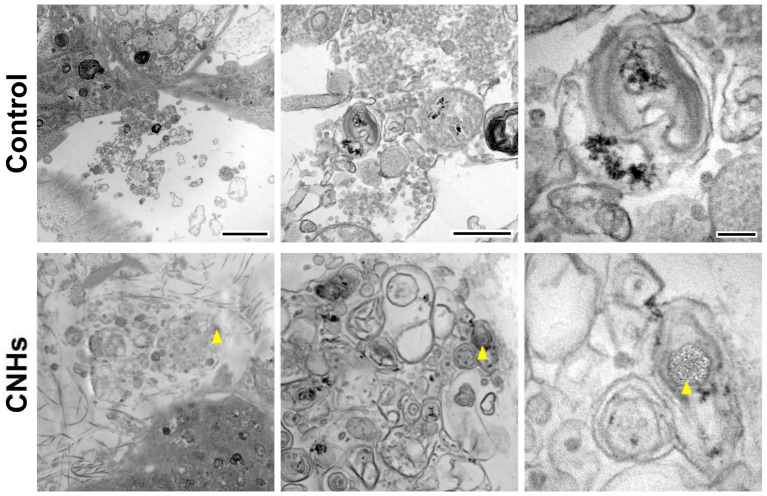
Localization of CNHs encapsulated in extracellular calcified nodules in the OB culture. OBs were exposed to CNHs under calcified conditions. Representative transmission electron microscope images with different magnifications are shown. Arrowheads indicate CNHs. Scale bars: 2 μm (**left** panels), 500 nm (**middle** panels), and 200 nm (**right** panels).

In order to assess the biological functions of osteoblasts with respect to important bone calcification, we evaluated the effects of CNHs under calcifying conditions. Alizarine red staining showed that the exposure of CNHs increased the reddish-purple stained calcified nodules compared with the control and CB exposure (Figure 7A). TEM images showed that CNHs were localized in calcified vesicles, and crystallization was observed on the surface of CNHs. In addition, CNHs were encapsulated in extracellular calcified nodules, and crystallization was observed within these vesicles (Figure 8). CB exposure appeared to have no effect on alizarin staining in the calcified OBs. TEM images showed that the CBs were localized in a sac-like structure that was different from that of the intracellular calcified vesicles (Figure 7B). Thus, there were differences in the subcellular localization of CNHs and CBs in addition to their status in TEM images after calcification.

### 3.6. Effect of CNMs on Bone Regeneration in Rat Tibia

**Figure 9:**
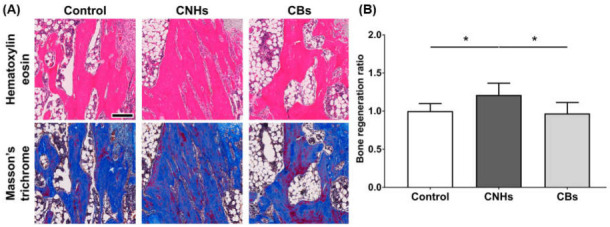
Effect of CNMs on bone regeneration. (**A**) Scale bar: 200 µm. The sections of tibial specimens of rats exposed to CNHs and CBs were stained with hematoxylin eosin and Masson’s trichrome staining. (**B**) Quantification of bone regeneration. New bone formation in bone tunnels was quantified using Masson’s trichrome staining images in (**A**). n = 6. Statistical significance was evaluated using the Tukey–Kramer test. * *p* < 0.05.

**Figure 10:**
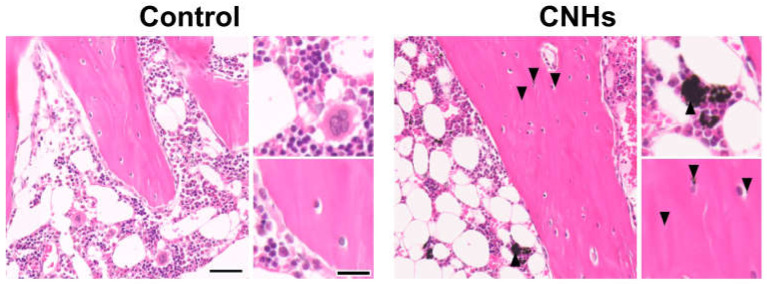
Distribution of CNHs in bone tissue. Representative image of tibial tissue specimens of the control and CNH rats stained with hematoxylin eosin. Arrowheads indicate CNHs. Scale bars: 2 µm (each **left** panels) and 500 nm (each **right** panels). Histological analysis was performed 12 weeks postoperatively.

Since experiments in vitro showed that CNHs alone affected bone-related cells, we decided to evaluate CNHs in vivo. In the bone regeneration of the rat tibia, the bone specimen images of red-pink HE staining and blue Masson’s trichrome staining showed increased fibrous bone tissues in the CNH group compared with the control and CB groups (Figure 9A). Quantitative evaluation of fibrous bone tissues near the bone tunnels, an area with the most prominent bone regeneration, also indicated that the area of bone regeneration in the CNH group was approximately 1.2 times greater than that in the control group (Figure 9B). In addition, the HE staining images in the CNH group showed uptake into macrophages in the bone marrow, in addition to accumulation of CNHs in the bone matrix and lacunae (Figure 10).

## 4. Discussion

In the present study, we used highly dispersed CNHs and CBs and performed in vitro and in vivo experiments to investigate their applications as DDS. As a result, we found that CNHs promoted wound healing in vivo without significant cytotoxicity, which indicates their usefulness in medical applications. Furthermore, CNHs inhibited the differentiation of OCs and promoted the differentiation and calcification of OBs in the in vitro experiments. As described in previous reports [18], we confirmed that differences in surface morphology affect cell response, even in CNMs of the same type. Although the cell uptake of CBs was observed in both OCs and OBs, the TRAP/ALP activity, differentiation marker gene expression levels, calcification, and bone regeneration were not observed. On the other hand, although CNHs showed a slight decrease in cell viability at the highest concentration, the highest concentration in this experiment was not considered cytotoxic according to the ISO10993-5 standard [22]. In addition, CNHs were incorporated into OCs and OBs, even when exposed to concentrations at which no cytotoxicity was observed. OCs showed decreased bioactivity and suppressed differentiation, whereas OBs showed increased bioactivity, promoted differentiation, and accelerated bone regeneration. In this experiment, CNMs were organized according to their weight ratios; therefore, it is possible that the difference in the number of particles during exposure may have had an effect. However, the difference in the state of CNMs encapsulated in calcified vesicles under the calcified conditions of OBs suggests that the cells may recognize the shape of CNMs, hence causing a different response.

In the current study, we clarified for the first time via in vitro experiments that CNHs suppress the bioactivity of OCs and cell differentiation. In a previous study, we revealed that CNTs, a type of CNM, are incorporated into osteoclasts; in addition, we showed that CNTs inhibit the differentiation signal of osteoclasts to suppress their differentiation [23]. Moreover, IL-6, an inflammatory cytokine, has been shown to act directly on osteoclasts, enhance the function of the macrophage system, and suppress differentiation into osteoclasts [24]. Furthermore, it has been reported that when CNHs were exposed to the macrophage-like cell line RAW264, a progenitor cell of OCs used in the present study, CNHs were taken up into the cells and increased the IL-6 dose dependently [17]. The present study clarified that exposure to CNHs at a concentration that does not cause cytotoxicity had decreased the TRAP activity of OCs. Real-time PCR analysis showed significantly decreased TRAP and Cathepsin K differentiation markers and increased IL-6 markers in the CNH group. These findings suggest that macrophages, which are progenitor cells during the differentiation process of OCs, may have taken up CNHs into the cells and released IL-6, thereby suppressing the bioactivity and differentiation of OCs.

Previous studies have shown that CNHs promote osteogenesis in vivo [19] and that CNHs are taken up by macrophages and oncostatin M, which are produced by the process to stimulate the differentiation of osteoblasts [20]. In the present study, the results of in vivo bone tissue specimens confirmed that CNHs were taken up by macrophages, suggesting that the mechanism shown in previous studies may have worked to promote bone regeneration. On the other hand, the ALP activity and differentiation marker genes increased in the CNH group in the present study as a result of direct exposure to CNHs in the OB culture alone. We also confirmed that aspects of cellular uptake of CNHs and CBs differed in the presence or absence of calcifying conditions. CNHs were localized in intra- or extracellular calcified vesicles and nodules under calcified conditions, and they had crystallized on the surface of the vesicles. Furthermore, in vivo bone tissue specimens confirmed that some CNHs were present in the bone matrix and within the lacunae. Moreover, CNHs resemble the shape of calcified vesicles [25]. These results suggest that OBs may have recognized CNHs as resembling the shape of calcified vesicles, incorporated them into the cells, and thus increased the number of calcified vesicles. Moreover, it is possible that CNHs acted as nuclei for crystallization within the calcified vesicles, increased the number of calcified nodules, and promoted bone regeneration.

The results of this study confirm that CNHs have low cytotoxicity and are effective as DDS for bone diseases. On the other hand, since CNHs affect physiological functions of cells and gene expression, it is necessary to select target diseases in which the existing environment is favorably affected by CNHs.

## 5. Conclusions

In the current study, we clarified the biological properties of CNHs alone on bone-related cells via in vitro and in vivo experiments. We also compared CNHs with CBs with a similar particle size but a different surface morphology. CNHs did not show severe cytotoxicity to bone-related cells, and they inhibited the bioactivity and differentiation of OCs, and promoted the bioactivity and differentiation of OBs. The in vivo experiments have shown that CNHs can effectively promote bone regeneration. We found that CNHs have high biocompatibility with bone tissue and could be developed as DDS, considering that CNHs affect the physiological activity, gene expression, and biological functions of the target disease.

## Figures and Tables

**Table 1:** Rheological size and zeta potential.

Materials	Rheological Size (nm)	Zeta Potential (mV)
CNHs	164.7 ± 54.0	−9.9 ± 1.6
CBs	220.2 ± 90.5	−10.8 ± 1.1

**Table 2:** List of target genes and primers for real-time PCR.

Takara Bio Primer_Set ID	Symbol	Name
MA050371	*Gapdh*	Glyceraldehyde-3-phosphate dehydrogenase
MA125620	*Acp5*	Acid phosphatase 5, tartrate resistant (ACP5)
MA115288	*Ctsk*	Cathepsin K
MA176482	*IL-6*	Interleukin-6
MA144435	*Runx2*	Runt-related transcription factor 2
MA147894	*Sp7*	Sp7 transcription factor (Osterix)
CH000874	*Bglap*	Bone gamma-carboxyglutamate protein (Osteocalcin)
MA127137	*Alpl*	Alkaline phosphatase, biomineralization associated

## Data Availability

All data are available from the corresponding author upon reasonable request.
